# Identification of Novel GSK-3β Hits Using Competitive Biophysical Assays

**DOI:** 10.3390/ijms23073856

**Published:** 2022-03-31

**Authors:** Beatrice Balboni, Shailesh Kumar Tripathi, Marina Veronesi, Debora Russo, Ilaria Penna, Barbara Giabbai, Tiziano Bandiera, Paola Storici, Stefania Girotto, Andrea Cavalli

**Affiliations:** 1 Computational and Chemical Biology, Istituto Italiano di Tecnologia, Via Morego 30, 16163 Genova, Italy; beatrice.balboni@iit.it (B.B.); shailesh.tripathi@iit.it (S.K.T.); 2 Department of Pharmacy and Biotechnology, University of Bologna, Via Belmeloro 6, 40126 Bologna, Italy; 3 D3 Pharmachemistry, Istituto Italiano di Tecnologia, Via Morego 30, 16163 Genova, Italy; marina.veronesi@iit.it (M.V.); debora.russo@iit.it (D.R.); ilaria.penna@iit.it (I.P.); tiziano.bandiera@iit.it (T.B.); 4 Structural Biology Laboratory, Elettra Sincrotrone Trieste S.C.p.A., Basovizza, 34149 Trieste, Italy; barbara.giabbai@gmail.com (B.G.); paola.storici@elettra.eu (P.S.)

**Keywords:** FBDD, drug discovery, NMR, cancer, Alzheimer’s disease

## Abstract

Glycogen synthase kinase 3 beta (GSK-3β) is an evolutionarily conserved serine-threonine kinase dysregulated in numerous pathologies, such as Alzheimer’s disease and cancer. Even though GSK-3β is a validated pharmacological target most of its inhibitors have two main limitations: the lack of selectivity due to the high homology that characterizes the ATP binding site of most kinases, and the toxicity that emerges from GSK-3β complete inhibition which translates into the impairment of the plethora of pathways GSK-3β is involved in. Starting from a 1D ^19^F NMR fragment screening, we set up several biophysical assays for the identification of GSK-3β inhibitors capable of binding protein hotspots other than the ATP binding pocket or to the ATP binding pocket, but with an affinity able of competing with a reference binder. A phosphorylation activity assay on a panel of several kinases provided selectivity data that were further rationalized and corroborated by structural information on GSK-3β in complex with the hit compounds. In this study, we identified promising fragments, inhibitors of GSK-3β, while proposing an alternative screening workflow that allows facing the flaws that characterize the most common GSK-3β inhibitors through the identification of selective inhibitors and/or inhibitors able to modulate GSK-3β activity without leading to its complete inhibition.

## 1. Introduction

Glycogen synthase kinase 3 beta (GSK-3β) is a serine-threonine kinase known for its ability to regulate glycogen synthesis and to play a critical role in many intracellular pathways, such as the signaling pathways involving insulin, growth factors, and neurothrophins (Wnt pathway and insulin pathway the most known) [1]. Moreover, it plays a critical role in regulating transcription factors controlling the expression of different genes [2].

In contrast with most kinases, GSK-3β is constitutively active, being inhibited in response to a specific stimulus, and it can only act on a pre-phosphorylated substrate [3]. In addition, GSK-3β is so adaptable that it can phosphorylate over 100 substrates [4], i.e., it is involved in sophisticated mechanisms that guarantee the phosphorylation of each substrate at specific times and subcellular compartments. Alteration of these mechanisms may lead to diseases. The self-activating nature that governs GSK-3β is, therefore, critical for the amplification of small GSK-3β deregulation to promote pathology [5,6].

The central role of GSK-3β in many intracellular pathways makes this enzyme an exciting and puzzling target for drug discovery. This widespread activity has also led to identifying GSK-3β dysregulation as responsible for the development of many diseases, such as Alzheimer’s disease (AD), bipolar disorder, diabetes, cardiovascular diseases, and cancer [7].

Promising GSK-3β inhibitors are already widely investigated for bipolar disorder [8,9], AD [10], inflammatory diseases [11], Acquired Immune Deficency Syndrome (AIDS) [12], diabetes [13,14] and cancer [15]. Moreover, according to the available literature data, GSK-3β inhibitors are in clinical trials for the therapy of AD [16] and Mild Cognitive Impairment (MCI) [17].

Nevertheless, a key issue encountered with GSK-3β inhibitors is the lack of selectivity due to the high degree of homology among the Adenosine TriPhoshate (ATP) binding pockets of several kinases. Moreover, the involvement of GSK-3β in multiple pathways was recently highlighted as a challenge from the drug discovery standpoint since a high degree of GSK-3β inhibition would suppress most of the paths in which the protein is involved, leading to toxicity [7].

It has been recently proposed that moderately weak inhibition of GSK-3β, enough to prevent self-activation, could be more appropriate since it would inhibit the amplification of a pathological related pathway while maintaining a partial GSK-3β activity in the non-pathological pathways involving the kinase [7].

In this work, to face and overcome these most common and critical flaws that characterize GSK-3β inhibitors, we set up an initial mono Dimensional Fluorine Nuclear Magnetic Resonance (1D ^19^F NMR) fragment screening in the presence of saturating concentrations of an ATP analog. The goal was the identification of GSK-3β inhibitors capable of binding to specific protein hotspots other than the ATP binding pocket or to the ATP binding pocket, but with an affinity able to compete with a selected reference binder. The competition with a reference binder allows setting an affinity cut-off during the initial fragment screening that would guarantee only the selection of compounds able to interfere with GSK-3β activity. A successive biophysical and structural characterization of the identified hits shows a prominent selectivity towards GSK-3β compared to other kinases that can be rationalized based on structural data. The setup pipeline is a promising workflow for the identification of allosteric and/or efficient GSK-3β inhibitors.

## 2. Results

To identify novel hit compounds able to inhibit GSK-3β, a subset of 350 fluorinated compounds (average MW of 270 Da) of an internal LEF (Local Environment of Fluorine) [18] compounds library was initially screened by ^19^F NMR binding experiments [19,20] against the target protein. The compounds were screened in mixtures of 20–25 each, through ^19^F transverse relaxation (^19^F T_2_) filter NMR experiments in the presence of saturating adenylyl-imidodiphosphate (AMP-PNP) concentrations (1 mM), a non-hydrolyzable ATP analog. Saturating AMP-PNP concentrations were selected based on previously performed GSK-3β AMP-PNP saturation experiments (data not shown).

**Figure 1:**
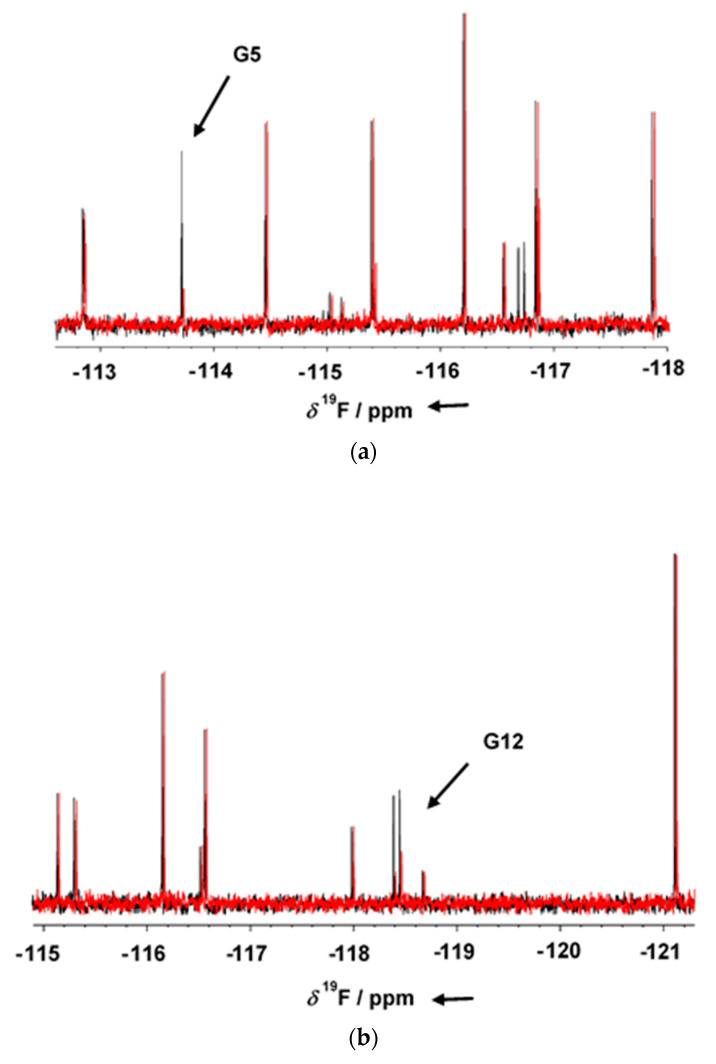
1D ^19^F T_2_ filter NMR spectra of fragment mixtures containing G5 (**a**) and G12 (**b**) fragments, in the presence of 1 mM AMP-PNP and in the absence (black) or presence (red) of GSK-3β. Binding compounds G5 and G12 give clear changes in their resonance signal in presence of protein (arrow). The ^19^F T_2_ filter spectrum of the two compounds was not affected by the presence of either 1 mM AMP-PNP.

**Figure 2:**
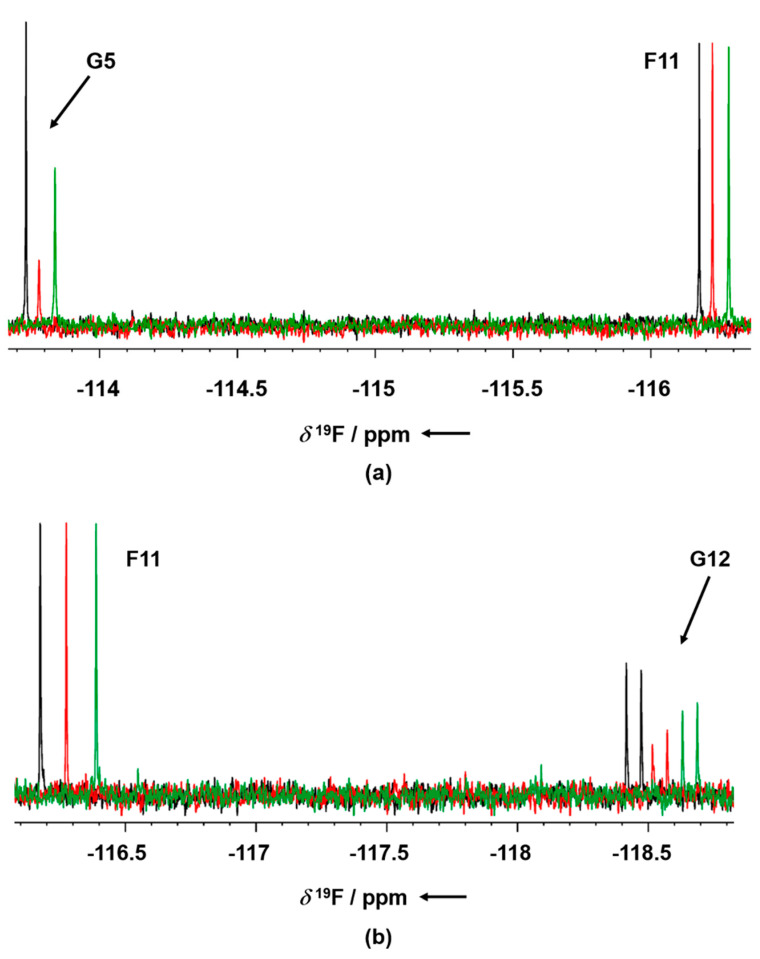
^19^F T_2_ filter NMR spectra of G5 (**a**) and G12 (**b**) in absence of GSK-3β (blue), in presence of GSK-3β and 1 mM AMP-PNP (red) and in the presence of GSK-3β and 3 mM AMP-PNP (green). F1 compound signal is used as the negative internal control. Binding compounds G5 and G12 show a strong line broadening of their NMR signals in the presence of the protein (arrow), whereas the NMR signal of the negative control (non-binder) remains unaltered in absence and in presence of protein. The ^19^F T_2_ filter spectrum of the two compounds was not affected by the presence of either 1 or 3 mM AMP-PNP.

GSK-3β was pre-incubated at room temperature for 10 min in the presence of 1 mM AMP-PNP before addition to the mixtures. Binding compounds (hits) were identified in the NMR screening by comparing ^19^F cpmg NMR spectra of each mixture in the absence and in the presence of the protein as shown in Figure 1a,b (black and red traces, respectively). The NMR signals of the molecules interacting with the protein showed a line broadening on their NMR signals in the presence of the protein, resulting in a reduction in their intensities. On the contrary, NMR signals of the non-binding molecules were not affected by the presence of the target protein. Molecules selected from this first NMR screening were then tested as a single compound under the same screening conditions in the presence of two non-binding compounds as negative controls. The promising compounds, still showing binding to GSK-3β when tested as singles, were retested also in the presence of 3 mM AMP-PNP (among these, G5 and G12, whose NMR spectra are reported in Figure 2a,b, respectively). Finally, 32 compounds still showed binding to GSK-3β also in the presence of higher AMP-PNP concentration. These hits were then further analyzed and characterized through different biophysical approaches.

Since all biophysical techniques normally tend to identify different sets of hits with sometimes little overlap we, therefore, decided to pursue hits identified by two biophysical techniques in parallel. The 32 compounds, identified through NMR, were further tested also in MST binding experiments where the movement of the fluorescently labeled target, in a temperature gradient, is recorded upon the addition of increasing concentrations of the hit compound under investigation.

**Figure 3:**
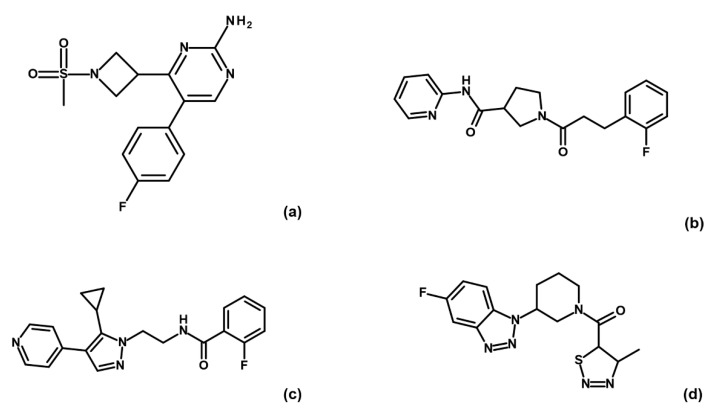
Chemical structures of fragments G5 (**a**), G12 (**b**), B3 (**c**) and E2 (**d**).

Binding check MST experiments were performed for the 32 compounds as direct binding or as displacement assays in the presence of 3 mM AMP-PNP (data not shown). Thirteen compounds showed a positive response in direct binding assays. Among these, only four hits (i.e., G5, G12, B3 and E2; Figure 3) maintained their ability to bind GSK-3β upon preincubation with AMP-PNP.

For these four hit MST experiments, increasing compounds concentrations were set up to determine the affinities of the compounds for the target protein.

**Figure 4:**
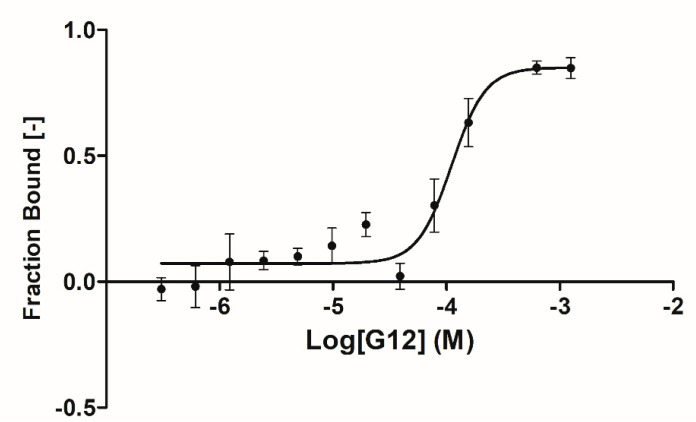
MST binding curve for G12 compound binding GSK-3β. Data are the average of three experiments (error bars denote Standard Deviation (SD)). Sigmoidal fitting was performed using the Affinity Analysis software of Nanotemper Technologies.

A complete binding affinity curve was obtained only for the G12 compound with an affinity parameter (k_d_) of 66.5 ± 31.8 µM (Figure 4). Solubility issues in the assay buffer encountered in testing the other three compounds (B3, E2 and G5) prevented us from obtaining a complete binding curve (high concentration plateau was missing) and, therefore, an affinity parameter. Further data analyses suggested that, for these three compounds, solubility issues may be coupled to an overestimation of the affinity parameters due to the steric hindrance induced by the fluorescent label bound to GSK-3β lysine residues (23 lysines are present in GSK-3β sequence).

Indeed, this suggestion was corroborated by MST experiments performed on staurosporine binding to GSK-3β, which provided an affinity parameter (k_d_) of ~67 nM (data not shown), compared to around 10 nM reported in the literature [21,22,23]. This information suggests that binding affinity data collected by MST may be partially affected by fluorescent labels steric hindrance that induces an overestimation of the detected affinity parameters.

**Figure 5:**
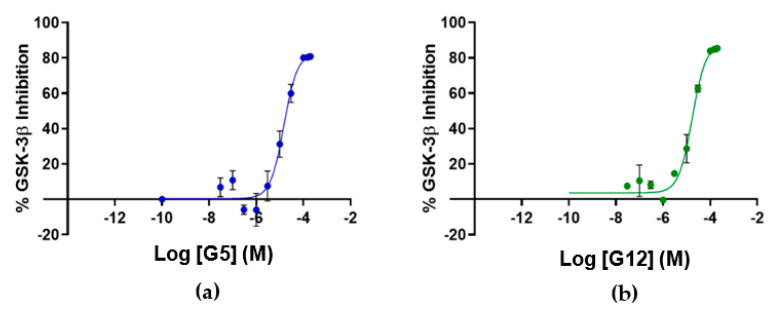
Dose-response curves for GSK-3β inhibition by G5 (**a**) and G12 (**b**). Error bars denote SD.

To assess if the identified GSK-3β binders were also able to affect kinase activity, the inhibitory activity of the four identified hit compounds was tested at four concentrations (1–5–50–100 μM) using the LANCE^®^ Ultra (Perkin Elmer, Waltham, MA, USA) time-resolved fluorescence resonance energy transfer (TR-FRET) assay. The assay measures the ability of GSK-3β to phosphorylate a specific substrate, human Muscle Glycogen Synthase (ULight-GS (Ser641/pSer657)), in the presence and absence of the identified ligands. While compounds B3 and E2 showed a poor ability to inhibit GSK-3β even at the highest concentration, G12 and G5 were selected for IC_50_ determination, since at 100 μM they showed more than 80% GSK-3β inhibition (Table 1). Specifically, IC_50_ values were (14.81 ± 0.55) μM and (15.25 ± 1.34) μM for G5 and G12, respectively (Figure 5).

To have insights into the mechanism of GSK-3β inhibition, G5 and G12 were tested for their ability to competitively replace ATP or the GSK-3β substrate ULight-GS (Ser641/pSer657), as described in the Section 4.

**Figure 6:**
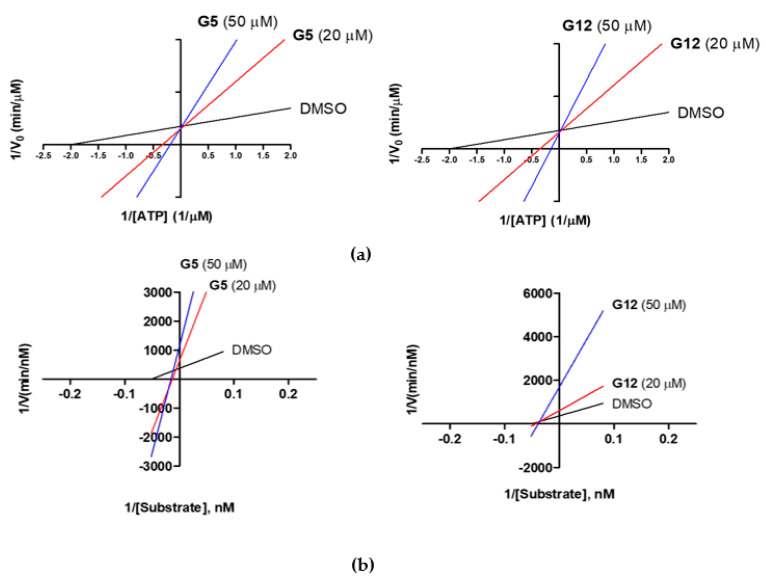
Lineweaver-Burk plots of GSK-3β kinetic data at two concentrations of G5 and G12 (20 and 50 μM). (**a**) Linear regression plotting of 1/V against 1/[ATP] at a given concentration of the compound. Intersecting at the same point on the y-axis indicates competitive inhibition with respect to ATP; (**b**) Linear regression plotting of 1/V against 1/[Substrate] at a given concentration of the compound. Intersecting at the same point on the x-axis indicates noncompetitive inhibition (G12), while for G5 the x intercept shifts right, suggesting a mixed inhibition with respect to the substrate.

First, under a constant concentration of the substrate ULight-GS (50 nM), ATP concentrations were varied from 0.25 to 4 μM, and G5 and G12 were tested at 20 and 50 μM. We observed an increase in the K_m_ constant (Michaelis-Menten constant), but an unaltered 1/V_max_ value, when the concentrations of G5 and G12 increased (Figure 6a), suggesting a competition between ATP and the two compounds. Additionally, G5 and G12 (at 20 and 50 μM) were tested by keeping the ATP concentration constant (1 μM) and varying the substrate ULight-GS concentration from 12.5 to 200 nM. At increasing concentrations, both compounds showed an increased 1/V_max_ value, but they differed in the effect on K_m_, which was unaltered for G12 while it increased for G5 (Figure 6b): this suggested that, in these experimental conditions, G12 did not compete with the substrate while G5 showed a mixed competition mechanism. Activity data, therefore, showed that despite the two selected molecules having very similar IC_50_s and being ATP competitive, G12 does not compete with the substrate. Conversely, G5 shows mixed competition towards the substrate.

Given the poor inhibitory activity of compounds B3 and E2, only G5 and G12 were selected for further biophysical and structural investigations. Thermal shift experiments, using SYPRO Orange fluorescent dye, able to bind the hydrophobic regions of denatured proteins, showed that, upon binding, both compounds induce an increase in the protein melting temperatures (T_m_), i.e., they tend to stabilize the structure of the protein (Supplementary Figure S1). A T_m_ increase of 3.2 °C and 2.5 °C was observed upon binding of G5 and G12, respectively. Reported effects were compared and normalized to the effect induced by DMSO alone on GSK-3β denaturation. The same experiments were also performed in the presence of AMP-PNP to unravel a possible effect of this molecule on protein denaturation. Notably, observed changes in T_m_ were not affected by the presence of AMP-PNP.

To further characterize the binding of the two identified compounds to GSK-3β, structural investigations were pursued through X-ray crystallography. Briefly, crystals of GSK-3β in complex with G5 or G12 were prepared by soaking compounds into the crystals of the protein in the apo form. Crystals were obtained after 1–2 days by the hanging drop vapor diffusion method (see Section 4), after buffer screening and optimization procedures. Structures of G5 and G12 in complex with GSK-3β were solved with the molecular replacement method at a 2.5 and 2.2 Å resolution, respectively (PDB 7U31, 7U2Z). Data collection and refinement statistics are presented in Supplementary Table S1.

**Figure 7:**
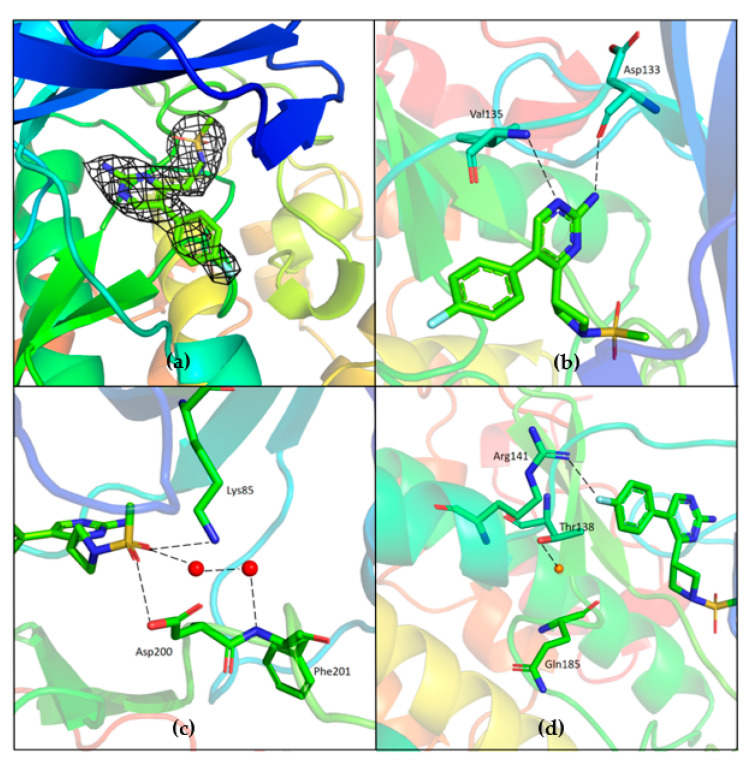
(**a**) Electron density corresponding to G5 contoured at 1.2 sigma; (**b**) Direct hydrogen bonding interaction between the core pyrimidine 2-amine of G5 with the backbone oxygen of Asp133 and nitrogen of Val135; (**c**) Hydrogen bonding interactions of methyl sulfonyl group of G5 with Lys85 and Asp200. Water (red spheres) mediated hydrogen binding network is also observed; (**d**) Conserved water (red sphere) is observed hydrogen-bonded to Thr138. Fluorine of fluoro-phenyl is hydrogen-bonded to Arg141; PDB 7U31.

**Figure 8:**
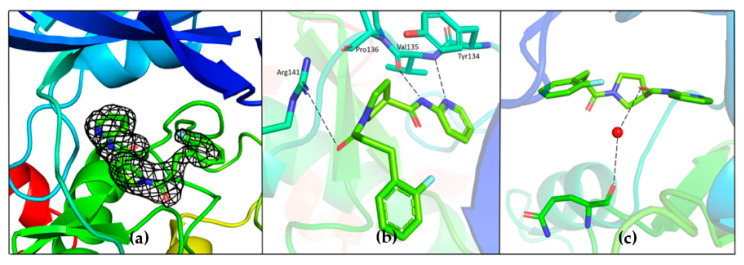
(**a**) Electron density corresponding to G12 contoured at 1.2 Sigma; (**b**) Hydrogen bonding interaction of G12 with the residues of hinge region; (**c**) Water-mediated hydrogen bond between carboxamide oxygen of G12 and Gln185; PDB 7U2Z.

Unambiguous electron density corresponding to both G5 and G12 was observed in the ATP binding pocket of GSK-3β (Figure 7a and Figure 8a).

**Figure 9:**
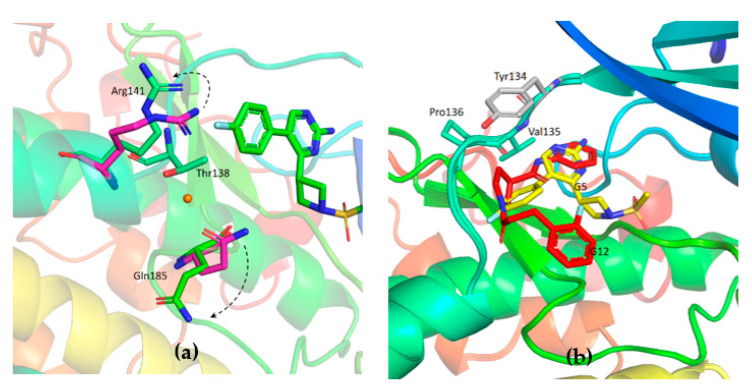
(**a**) In GSK-3β-G5 crystal complex (green), residues Arg141 and Gln185 are flipped away from the binding pocket in comparison to PDB 1Q4L (purple); (**b**) Overlapped structures of G12 (red) and G5 (yellow).

G5 interactions with protein residues are reminiscent of GSK-3β specific maleimide analogs. The methyl sulphonyl group of G5 interacts with Lys85 and Asp200 sidechains via hydrogen bonding interactions (Figure 7c). The core pyrimidine 2-amine is involved in a direct hydrogen bonding interaction with the backbone oxygen of Asp133 and nitrogen of Val135 (Figure 7b). The conserved water molecule next to Thr138 is observed close to the putative fluoro-phenyl site (Figure 7d). The fluorine atom of the fluoro-phenyl portion of G5 is involved in a direct hydrogen bond with Arg141 (Figure 7d). Gln185 and Arg141 are flipped away from water (Figure 9a), while in the published structures of GSK-3β in a complex with a maleimide derivative, they are hydrogen-bonded to the conserved water molecule and to the compound itself (PDB: 1Q4L).

The binding position of G12 in the ATP pocket completely differs from the one of G5. Electron density corresponding to G12 is reported in Figure 8. Direct hydrogen bonding interactions are observed between pyridine nitrogen and carboxamide nitrogen with the backbone atoms of Tyr134 and Val135, respectively (Figure 8b). The propanoyl oxygen of G12 is involved in a hydrogen bonding interaction with Arg141 (Figure 8b). Electron density corresponding to the fluoro-phenyl group is not very well defined, suggesting that this portion of the G12 molecule is lacking adequate stabilizing interactions with the protein. A water-mediated hydrogen bond network is observed between the carboxamide oxygen of G12 and the backbone oxygen of Gln185 (Figure 8c). A similar interaction has been already observed and proposed to justify the specificity of ruthenium based inhibitors against GSK-3β [24].

**Figure 10:**
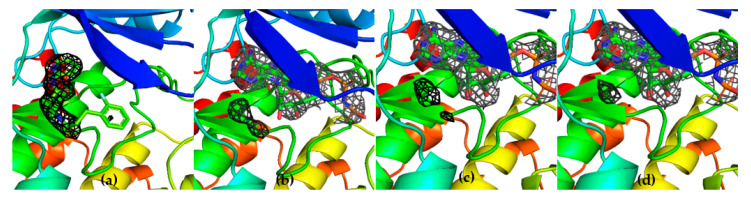
Three-dimensional structures GSK-3β in complex with G12 in the presence of increasing concentrations of AMP-PNP (0 mM AMP-PNP (**a**); 1 mM AMP-PNP (**b**); 2 mM AMP-PNP (**c**); 4 mM AMP-PNP (**d**)).

To further confirm the competitive behavior of the two fragments with AMP-PNP, crystals of GSK-3β in the presence of G12 and increasing concentrations of AMP-PNP were grown. Collected electron density data confirmed the ATP competitive behavior of G12, showing the compound being progressively displaced from the ATP-binding site in an AMP-PNP dependent fashion (Figure 10).

To further characterize the inhibitory activity of the two selected fragments, the selectivity of G5 and G12 towards a panel of 58 kinases of the humane proteome was evaluated using the “Kinase screening and profiling” service of Eurofins, Luxembourg (Kinase Diversity Panel). Inhibitory activity assays were performed at constant compounds concentration (20 µM) and at an ATP concentration, specific for each kinase (K_m_ ± 15 µM). Results are reported in Supplementary Figures S2 and S3.

Both G5 and G12 compounds were shown to be, on average, more active on GSK-3β than on most of the other kinases screened in the panel. Activity comparable to the one observed on GSK-3β was detected only in less than 10% of the screened proteins. Notably, among the other kinases, G5 showed a significant inhibitory effect also on p70S6K (26%), SAPK2a (19%), LOK1 (21%) and G12 also showed a significant inhibitory effect on CDK9 (50%), TAK1 (20%), p70S6k (15%). These cross-reactivity results provided new information on inhibitors’ binding ability and suggested the existence of common structural and binding features among the structures of these kinases-inhibitor complexes.

**Figure 11:**
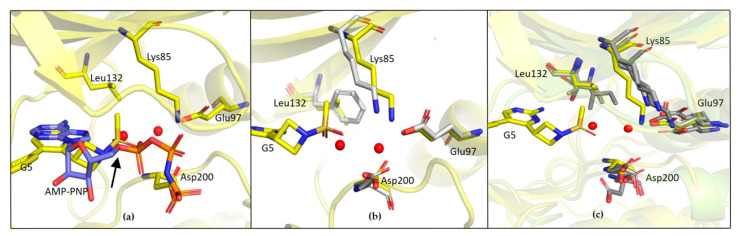
(**a**) Overlapped structure of GSK3β-G5 complex with GSK3β-AMP complex (PDB 1PYX); the black arrow indicates the first phosphate of AMP-PNP (**b**) Overlapped structure of GSK3β-G5 with CDK9 structure (PDB 3BLQ); (**c**) Overlapped structure of GSK3β-G5 with kinases showing inhibition with G5 (P70S6K/PDB 3A61, SAPK2a/PDB 1OZ1 and LOK1/PDB 6HXF). GSK3β-G5 complex is in yellow and comparison structures are represented in grey.

The observed specificity of G5 towards GSK-3β was initially ascribed to its interactions with Lys85, Asp200 and Arg141 based on structural comparisons and a previous study by Kramer and colleagues, which claimed that the residues Lys85, Asp133, Val135, Glu137, Arg141, Gln185, Asp200 and Arg220 are crucial amino acids for interactions with the binding pocket of GSK-3β [25]. In fact, kinases inhibited by G5 to a similar extent as GSK-3β (i.e., P70S6K, SAPK2a and LOK1) have conserved Lys85 and Asp200 residues. Surprisingly, kinases whose activity was only marginally affected by G5 (such as AMPK1α and MEK1) conserve Lys85 and Asp200 in the same positions. This analysis suggests that the presence of these residues might not be the explanation for G5 selectivity. The structure of GSK-3β-G5 was also overlapped with the available GSK-3β-AMP-PNP structure (PDB 1PYX). Here, relevant proximity of the methyl sulphonyl group of G5 to the first phosphate of AMP-PNP is visible (Figure 11a, black arrow, suggesting that G5 inhibits GSK-3β acting as ATP. Moreover, from the kinase panel, G5 did not show any evident inhibitory activity on CDKs (Supplementary Figures S2 and S3), even though these kinases have high structural homology with GSK-3β (86% structure homology [26]). Leu132 in GSK-3β is replaced by a bulkier phenylalanine in all the CDKs (Figure 11b). In GSK-3β, water molecules next to Leu132 are involved in a hydrogen bonding network with the methyl sulphonyl group of G5 and Lys85, Asp200. When in CDKs Leu132 is replaced by a bulkier phenylalanine, this network is disrupted (Figure 11c). Indeed, the presence of Leu132 and the related water-mediated hydrogen bonding network could be the explanation for G5 selectivity on GSK-3β activity.

**Figure 12:**
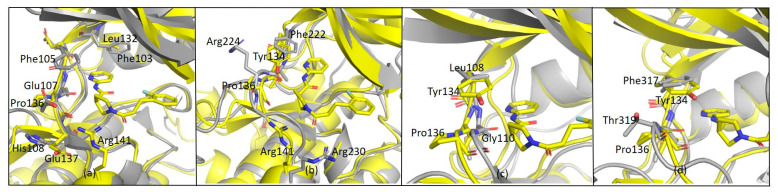
(**a**) Overlapped structure of CDK9 (PDB 3BLQ) and GSK3β-G12 complex; (**b**) Overlapped structure of GSK3β-G12 and MLK1 (PDB 4UY9); (**c**) Overlapped structure of GSK3β-G12 and SAPK2A (PDB 4EWQ); (**d**) Overlapped structure of GSK3β-G12 and ABL (PDB 4WA9).

As far as G12 is concerned, this compound is more effective on CDK9 and TAK1 kinases rather than on GSK-3β, while other kinases (i.e., MLK1, SAP2a and ABL1) are almost unaffected by the presence of the small molecule. CDK9 and GSK-3β have a sequence homology of 48% and very similar structures (Figure 12a). However, according to structure overlap, the presence of Phe103 in CDK9 may lead to a shift of G12 along the hinge and to the formation of a new π-stacking interaction between the pyrimidine ring of G12 and the phenylalanine ring moiety. This interaction could explain the ability of G12 to inhibit CDK-9 rather than GSK-3β.

Other considerations were made comparing MLK1 (i.e., MAP3K9) and GSK-3β-G12 structure. MLK1 shares 47% of sequence homology with GSK-3β. Arg141 in GSK-3β is retained as Arg230 in MLK1 (Figure 12b), even though no inhibition was reported. In addition to this, in the MLK1 structure, Pro136 of GSK-3β is replaced by Arg224. This evidence suggests that Arg141 is not a key residue for GSK-3β-G12-interaction and shows that the relatively restrained backbone of the hinge region offered by Pro136 is critical for G12 binding to GSK-3β. In line with these data, Pro136 is replaced by Gly110 in SAPK2a (Figure 12c) and by Thr318 in ABL (Figure 12d): in these kinases, no inhibitory effect was observed in the presence of G12. Interestingly, Pro136 is replaced by Glu107 also in CDK9. However, in CDK9, since G12 might be shifting upwards to have a stacking interaction with Phe103, its interaction with Pro136 could not be necessary, as a new backbone of hydrogen bonds might be provided by Cys106, Phe105, or Asp104. Overall, backbone interactions offered by Pro136 seem more relevant in G12 binding to GSK-3β compared to Arg141 side-chain interactions. G12 might, therefore, be not only a good initial hit to be developed as a GSK-3β inhibitor but also a good starting point for the development of specific inhibitors for CDK9/Cyclin T1, which is already known as a pharmacologically important target [27].

In this work starting from a ^19^F NMR fragment screening, two fragments able to bind the ATP binding site of GSK-3β were identified and characterized through biophysical assays. Structural characterization further assessed their binding poses and ability to selectively inhibit GSK-3β rather than other kinases.

**Figure 13:**
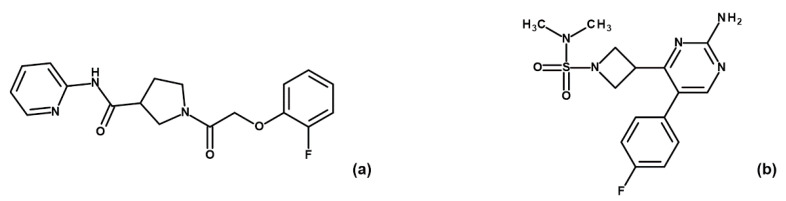
Chemical structures of fragments ARN1484 (**a**) and ARN9133 (**b**).

**Figure 14:**
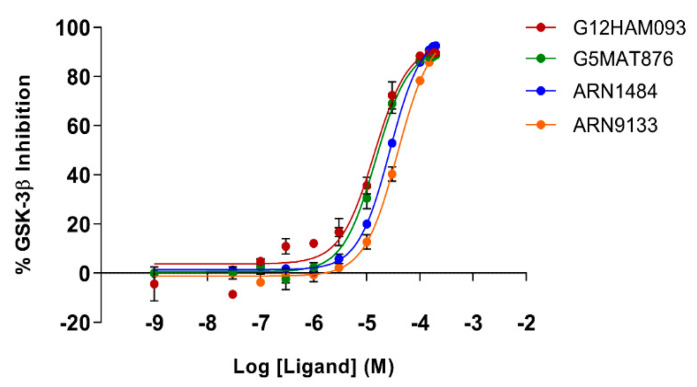
Dose-response curves for GSK-3β inhibition by ARN1484 (blue), ARN9133 (orange), G5 (green) and G12 (red). Error bars denote SD.

**Figure 15:**
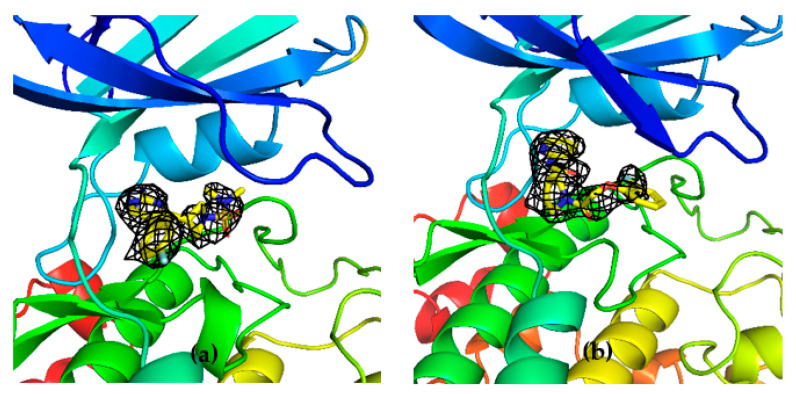
(**a**) 3D structure of GSK-3β in complex with ARN9133 (PDB 7U33); (**b**) 3D structure of GSK-3β in complex with ARN1484 (7U36).

To corroborate our structural data, we performed an additional computational homology search of our internal database to identify G5 and G12 homologous compounds. The experimental analysis of the retrieved homologs was performed in the attempt to gather more details about binding affinities and residue contributions and, eventually, identify more potent compounds. Out of seven identified homologous, two compounds, ARN1484 and ARN9133 (Figure 13), showed a similar inhibitory activity (IC_50_) to G5 and G12 (38.1 µM and 27.2 µM, respectively; Figure 14). The 3D crystal structures of these compounds in complex with GSK-3β were solved (Figure 15). Crystal structures of ARN1484/ARN9133-GSK-3β (PDB 7U36, 7U33) complexes showed a network of interactions for these two compounds with the protein very similar to the ones observed for G5 and G12, corroborating their similar IC_50_ values.

## 3. Discussion

In this work, an NMR screening campaign of an IIT fluorinated fragments library allowed the identification of hit compounds able to bind GSK-3β in the presence of saturating AMP-PNP concentrations. The fragment-based approach was chosen to pursue selectivity, exploiting the idea that small molecules should better fit into proteins pockets. Nevertheless, small molecules are frequently weak binders that need to be evolved into more complex molecules to become efficient binders. Running the screening in the presence of AMP-PNP, allowed us to directly exclude too weak binders and compounds that, by not being able to compete with an ATP analog, will hardly be able to affect GSK-3β activity.

Moreover, running the initial screening in the presence of AMP-PNP saturating conditions allowed to reduce, from the beginning of the process, the number of hits–narrowing down the initial screening campaign.

The compounds identified from our initial NMR screening on GSK-3β were tested with MST to confirm the binders and further narrow down the number of hits: indeed, only four compounds among the 32 molecules chosen by NMR showed binding to GSK-3β, not only in the absence of AMP-PNP but also under AMP-PNP saturating concentrations. These four compounds were further characterized since their ability to bind GSK-3β also in the presence of saturating AMP-PNP concentrations was interpreted as the possibility to bind the enzyme in multiple pockets including the ATP binding site.

Activity assays were further performed to better characterize the kinetic properties of the binding and to obtain insights into the mechanism of inhibition. G5 and G12 showed a good GSK-3β inhibitory activity (more than 80% at 100 μM concentration) and were selected for further investigations. G5 and G12 binding to GSK-3β was corroborated by thermal shift assays where the binding of the fragments to the protein, induced a stabilizing effect compared to the protein alone. Indeed, from displacement assays, G12 is an ATP-competitive inhibitor not substrate competitive, while G5 is an ATP-competitive inhibitor of GSK-3β with a mixed inhibition for the substrate.

The two identified compounds were then tested against a panel of kinases for their ability to inhibit kinase activity. Both G5 and G12 turned out to be rather selective for GSK-3β relative to other kinases. Notably, G12 showed higher activity on CDK9 compared to GSK-3β.

The 3D structures solved for GSK-3β in complex with G5 and G12 showed that the main interactions between the compounds and the protein could be traced to the ones observed for other already known ATP-competitive GSK-3β inhibitors.

In a further attempt of explaining the observed specificity of the identified compounds for GSK-3β, the available 3D structures of a few kinases, present in the selectivity panel study, were compared and overlapped with the structure of GSK-3β in complex with G5 and G12. This analysis allowed us to putatively ascribe the selectivity of the compounds observed for GSK-3β to the formation of hydrogen bonding networks that are peculiar to the interaction of these two molecules with the protein. This hypothesis was corroborated by activity data and 3D structures collected and solved for two analogs in complex with GSK-3β.

Despite the importance of GSK-3β as a key target for several pathologies and the very high number of inhibitors that have been proposed over the years, the progression of these molecules to the clinical stage is extremely slow due to several challenges mainly related to off-target activity and toxicity [28]. To address these issues common to all protein kinases, the drug discovery research has been moved towards the search for allosteric inhibitors to identify molecules able to bind and inhibit the target with high selectivity [29,30,31]. The search for allosteric druggable pockets (other than the ATP binding site) for identifying highly selective GSK-3β inhibitors has been extensively carried out [32,33,34,35]. Nevertheless, not much progress has been made towards the clinical stage.

In addition, for GSK-3β the high toxicity and side effects observed in the clinical phase have been ascribed not only to the lack of inhibitors selectivity but also to the involvement of this target in multiple pathways: a strong inhibition of GSK-3β suppresses most of the pathways, pathological or not, in which the protein is involved with a strong impact on cell activities and survival [7].

Since the activity of GSK-3β is slightly higher than its normal activity under pathological conditions, a moderate inhibition has been recognized as the best approach to inhibit GSK-3β correcting aberrant kinase behavior and preserving its participation in the non-pathological pathways [36,37].

To overcome these difficulties encountered in unveiling GSK-3β inhibitors, we thus designed a workflow not aiming at the high inhibitory potency of molecules, but instead selectivity to avoid a total inhibition of all the pathways in which GSK-3β is involved and limit side effects when translated into the clinic. Our approach successfully allows us to discover selective GSK-3β inhibitors, which, even if ATP-competitive, show good GSK-3β selectivity and a mid-weak potency. These molecules represent a promising starting point for the development of novel GSK-3β inhibitors. If the good selectivity towards other kinases is conserved in more potent analogs, they will most likely have fewer side effects. It remains to be ascertained which is the optimal potency in the inhibition of GSK-3β that combines a specific therapeutic activity with a limited impact on other pathways that the kinase is involved in. 

## 4. Materials and Methods

### 4.1. Expression and Purification of Recombinant GSK-3β

Phosphorylated GSK-3β was expressed in Sf9 cells and purified according to the protocol published by Gobbo and colleagues in 2019 [38]. Cell pellets of 900 × 10^6^ cells were resuspended and thawed in lysis buffer (20 mM TRIS pH 8, 0.5 M NaCl, 10 mM Imidazole, 1 mM DTT, 5 mM MgCl_2_, 0.5× protease inhibitor EDTA free (Roche, Basel, Swiss), 5% glycerol, 0.01% Tween20) and lysed with a sonicator (12′ pulse at 60–70% intensity). After sonication, the lysate solution was incubated for 20 min at 4 °C with DNAse I 5 ug/mL (MilliporeSigma, Burlington, MA, USA), then centrifuged for 1h at 4 °C at 30,000× *g*. After centrifugation, the supernatant solution containing the protein of interest underwent a two-step-purification procedure. First, the clarified supernatant was incubated for 2h with Ni-NTA agarose resin (Qiagen, Hilden, Germany) and washed out with a binding buffer containing 10 mM Imidazole (20 mM TRIS pH 8, 0.5 M NaCl, 10 mM Imidazole, 5% glycerol, 1 mM DTT). Elution was performed with buffer containing 0.3 M imidazole (20 mM TRIS pH 8, 0.5 M NaCl, 0.3 M Imidazole, 5% glycerol, 1 mM DTT). Eluted proteins were then diluted in 20 mM Hepes and 1 mM DTT only, to reach the correct salt concentration of ~40 mM NaCl in order to load it on a cationic exchange column *HiTrap HP SP (Cytiva)* (loading buffer: 20 mM Hepes pH 7.5, 30 mM NaCl, 5% glycerol, 1 mM DTT). At this point, the protein was purified and cleaned from solution impurities with a step gradient elution (elution buffer: 20 mM Hepes pH 7.5, 1 M NaCl, 5% glycerol, 1 mM DTT), first with 8% elution buffer (washing step), then with 13%, and, finally, increasing the elution buffer concentration up to 100%, allowing all GSK-3β isoforms to be eluted. Only the first peak of the chromatography eluate, at 100–130 mM NaCl, corresponding to phosphorylated (pTyr216) and most active GSK-3β isoform, was used for further experiments [39]. Obtained protein aliquots (5–15 µM concentration) were collected and stored at −80 °C.

### 4.2. ^19^F NMR Ligand-Based Binding Screening

All NMR screening experiments were recorded at 298 K with a Bruker FT NMR Avance III 600 MHz spectrometer, equipped with a 5 mm CryoProbe™ QCI ^1^H/^19^F–^13^C/^15^N–D with an automatic sample changer SampleJet™ with temperature control. All GSK-3β experiments were performed at low enzyme concentration (750 nM/1 μM) in 60 mM Hepes pH 7.5, 25 mM NaCl, 10 mM MgCl_2_, 2 mM TCEP, 8% D_2_O (for lock signal), 0.003% Triton X-100 (for the coating of NMR tube wall). About 350 fluorinated fragments, belonging to the internal LEF library were screened at 40 μM in mixtures of 20–25 compounds each, in the presence of 1 mM AMP-PNP (Sigma Cat. Numb. A2647) and in the absence (control) or presence of 1 μM GSK-3β.

Promising hits were tested as single compounds in the presence of two non-binders (negative controls) under the same experimental screening conditions to further confirm the bindings observed in mixtures. Bindings of the confirmed hits were further analyzed by testing the compounds at 20 μM in the absence or the presence of 750 nM GSK-3β and in the absence or the presence of saturating concentrations of AMP-PNP (1 mM and 3 mM). For each sample 1D ^19^F with ^1^H experiment and ^19^F T_2_ filter experiments were recorded with the Carr–Purcell–Meiboom–Gill (cpmg) scheme [40,41] with a 23.5 ms time interval between the 180° pulses and different total length (94, 188, 282 and 376 ms, respectively). All NMR experiments were run with proton decoupling using the Walts 16 composite pulse sequence with a 90° pulse of 120 ms, a spectral width of 50 ppm, acquisition time of 0.58 s, relaxation delay of 5 s and 128 scans. Spectra were transformed using a line broadening of 1 Hz before Fourier transformation. All fluorine chemical shifts were referred to as the signal of CFCl_3_ in water.

### 4.3. MicroScale Thermophoresis

MicroScale Thermophoresis (MST) experiments were conducted in triplicate on a Monolith NT.115 Pico system (NanoTemper Technologies, Munich, Germany).

The MST technique was used both for hit binding and for affinity (k_d_) determination.

GSK-3β was labeled with the His-tag RED-tris-NTA label 2nd generation (Nanotemper Technologies) following Nanotemper technology protocol. The 100 nM stock labeled protein solution was used at 20 nM concentration to roughly start from 7000 fluorescence counts in each screening experiment.

GSK-3β was labelled also using RED-NHS ammine dye (Nanotemper Technologies) following the manufacturer’s protocol. The obtained ammine-labeled protein was used at 20 nM concentration to roughly start the experiments from 6000 fluorescence counts.

The highest concentration tested for compounds binding (in the presence or absence of AMP-PNP saturating concentrations) was between 200 and 400 μM, depending on compounds solubility in the assay buffer. Binding tests were performed on eight capillaries with a constant concentration of the labeled protein (20 nM), to detect the effect induced by the compounds on the fluorescence signal (in the presence or absence of 3 mM AMP-PNP). Protein in assay buffer in the presence of 5% DMSO was the negative control. For binding affinity assays, MST measurements were performed on 16 capillaries with a constant concentration of labeled protein and 16 increasing concentrations of the compound in the absence or presence of saturating conditions of AMP-PNP (3 mM).

Assay buffer: Phosphate buffer 0.1 M pH 7.2, 5 mM MgCl_2_, 0.001% Tween80, 0.1% PEG8000. Experiments were performed in triplicates. Data elaboration and plots were performed using MO.Control Nanotemper software.

### 4.4. GSK-3β Activity and Inhibition Assays

The GSK-3β kinase assay was run in 384 well microplates (OptiPlate^TM^-384, White, Perkin Elmer) with a total reaction volume of 20 µL. The inhibitory potency against human recombinant GSK-3β was evaluated using the LANCE^®^ Ultra (Perkin Elmer) time-resolved fluorescence resonance energy transfer (TR-FRET) by measuring the phosphorylation of the specific substrate, human Muscle Glycogen Synthase (ULight-GS (Ser641/pSer657)), according to the manufacturer’s instructions. For the screening and dose-response curves, test compounds, staurosporine (reference compound) or DMSO (control) were mixed with the enzyme (2 nM) in a buffer containing 50 mM Hepes (pH 7.5), 1 mM EGTA, 10 mM MgCl_2_, 2 mM DTT and 0.01% Tween20. The reaction was initiated by adding 50 nM of the substrate ULight-PASVPPSPSLSRHSSPHQ(pS)ED and 1 µM ATP. The mixture was incubated for 1 h at 23 °C. Afterward, the reaction was stopped by adding 8 mM EDTA. After 5 min, the anti-phospho-GS antibody labeled with europium chelate was added. 1 h later, the kinase reaction was monitored by irradiation at 320 nm, and the fluorescence was measured at 615 and 665 nm, using EnVision 2014 Multilabel Reader (PerkinElmer). The calculated signal ratio at 665/615 nm was proportional to the extent of ULight-GS phosphorylation. The compounds were screened at four concentrations (1–5–50–100 μM). For selected compounds, dose–response curves, ranging from 30 nM up to 200 μM, were performed. Dose-response curves were run in three independent experiments, each performed in three technical replicates. IC_50_ values (concentrations causing half-maximal response or enzyme inhibition) were determined by non-linear regression analysis of the Log [concentration]/response curves generated with mean replicate values using a four parameter Hill equation curve fitting with GraphPad Prism 8 (GraphPad Software Inc., San Diego, CA, USA).

To study the GSK-3β kinetics, the reaction mixture, varying concentrations of ATP (0.25–0.5–1–2–4 μM) or substrate (12.5–25–50–100–200 nM) versus test samples (20 and 50 μM), were incubated for 5, 15, 30, and 60 min at 23 °C, followed by the addition of the 6 mM EDTA and the anti-phospho-GS antibody according to the manufacturer’s protocol.

For GSK-3β kinetic experiments, initial velocities (V_0_) were determined and fitted to the Michaelis–Menten equation. To directly visualize G5 and G12 inhibition mode, a Lineweaver–Burk plot was generated according to the values obtained from the Michaelis–Menten analysis. The slope corresponded to K_m_/V_max_, the intercept on the vertical axis to 1/V_max_, and the intercept on the horizontal axis to −1/K_m_. Moreover, at the reciprocal of the smallest value of substrate concentration (X = 1/[S_min_]) was associated a value of Y representing the equation Y = (1/V_max_)(1.0 + K_m_/[S_min_]). Graphs and data analysis were performed using GraphPad Prism 8 software.

### 4.5. Thermal Shift Assays

Thermal shift experiments were conducted in technical triplicates and recorded with a *ViiA7^TM^* real-time PCR instrument. The analysis protocol of Partch and colleagues developed for fast stability screening of buffers and interactors for recombinant proteins was followed, [42]. Briefly, the recombinant protein GSK-3β at a final concentration of 5 μM was mixed with the dye SYPRO Orange (2× final concentrated) in the selected assay buffer (NaCl 300 mM, Hepes pH 7.5 20 mM, MgCl_2_ 5 mM, DTT 1 mM) and incubated with hit compounds at two different concentrations, 200 and 500 μM. Experiments were also performed on GSK-3β also in the presence of AMP-PNP at a final concentration of 200 μM. A 96-well plate was prepared with samples in triplicate; samples were prepared in total reaction volumes of 50 μL (protein concentration 5 μM and compound 200 μM); 2% DMSO was the final concentration in each sample. Plates were then centrifuged at 800g for 2 min at 25 °C. As soon as the plate was placed into the Applied Biosystems ViiA7 real-time PCR instrument a protocol was run setting a melting curve from 25 to 95 °C with the step and hold run method.

Output data were collected and analyzed with Excel software and Graphpad Prism 7.

### 4.6. Protein Molecule Complex Crystallization and X-ray Data Collection

Purified GSK-3β protein was concentrated to 3–4 mg/mL. Crystals were grown with the hanging drop vapor diffusion method, using 20 mM Hepes pH 7.5, 50 mM MgCl_2_ and 15–20% PEG3350 as crystallization buffer. Protein crystals appeared within 1–2 days. Apo crystals were soaked in a solution, which was constituted of the same crystallization buffer supplemented with 6–8% glycerol and 1 mM final concentration of G5, G12, ARN1484, ARN9133. These crystals were frozen in liquid nitrogen, after 5–6 h of soaking.

Data collection was performed at the XRD2 beamline of Electra Synchrotron in Trieste, Italy. Data reduction was performed using iMOSFLM [43], scaling was performed using AIMLESS [44] and refinement was performed using REFMAC [45]. Ligand drawing was performed using a ligand in a CCP4 suit. Model modification and visualization were performed using Coot [46]. Images were finally constructed using Pymol [47]. Refinement values are reported in Supplementary Table S1.

## Figures and Tables

**Table 1:** %inhibition of GSK-3β obtained for G5, G12, B3 and E2.

		%Inhibition (*)		
	G5	G12	B3	E2
1 μM	0.3 ± 0.1	8.5 ± 0.1	2.5 ± 0.9	ni
5 μM	13.4 ± 2.0	15.2 ± 2.7	4.9 ± 1.5	ni
50 μM	69.4 ± 2.0	71.4 ± 1.7	27.1 ± 4.9	8.2 ± 1.9
100 μM	84.5 ± 0.4	88 ± 1.2	46.6 ± 6.0	18.2 ± 1.5

(*) data are expressed as mean value ± SD.

## Data Availability

The atomic coordinates and structural factors of GSK-3β in complex with G5, G12, ARN1484 and ARN9133 have been deposited in the Protein Data Bank (PDB) with the accession number PDB: 7U31 (GSK-3β in complex with G5); 7U2Z (GSK-3β in complex with G12); 7U36 (GSK-3β in complex with ARN1484); 7U33 (GSK-3β in complex with ARN9133).

## Supplementary Materials

The following supporting information can be downloaded at: https://www.mdpi.com/article/10.3390/ijms23073856/s1.
