# Supplementary material for: Identification of Novel GSK-3β Hits Using Competitive Biophysical Assays
Source: Int J Mol Sci. 2022 Mar 31;23(7):3856. doi: 10.3390/ijms23073856 (PMC8998611; doi:10.3390/ijms23073856)
Supplement: Supplementary file 1 [file ijms-23-03856-s001.zip › ijms-1641081-supplementary.pdf]

SUPPLEMENTARY INFORMATION

Identification of novel GSK-3β hits using competitive biophysical assays

Beatrice Balboni[1,2], Shailesh Kumar Tripathi[1] , Marina Veronesi[3], Debora Russo[3], Ilaria Penna[3], Barbara Giabbai[4], Tiziano Bandiera[3], Paola Storici[4], Stefania Girotto[1] and Andrea Cavalli[1,2]**

[1]*Computational and Chemical Biology, Istituto Italiano di Tecnologia, Via Morego 30, 16163 Genova, Italy*

[2]*Department of Pharmacy and Biotechnology, University of Bologna, Via Belmeloro 6, 40126 Bologna, Italy.*

[3]*D3 Pharmachemistry, Istituto Italiano di Tecnologia, Via Morego 30, 16163 Genova, Italy.*

[4]*Structural Biology Lab, Elettra Sincrotrone Trieste S.C.p.A., 34149, Basovizza, Trieste, Italy*

*Andrea Cavalli, Computational and Chemical Biology, Istituto Italiano di Tecnologia, Via Morego 30, 16163 Genoa, Italy, phone: +39 0102897403; Stefania Girotto, Computational and Chemical Biology, Istituto Italiano di Tecnologia, Via Morego 30, 16163 Genoa, Italy, phone: +39 0102896983

Email: andrea.cavalli@iit.it, stefania.girotto@iit.it

Supplementary figures

Supplementary Figure S1

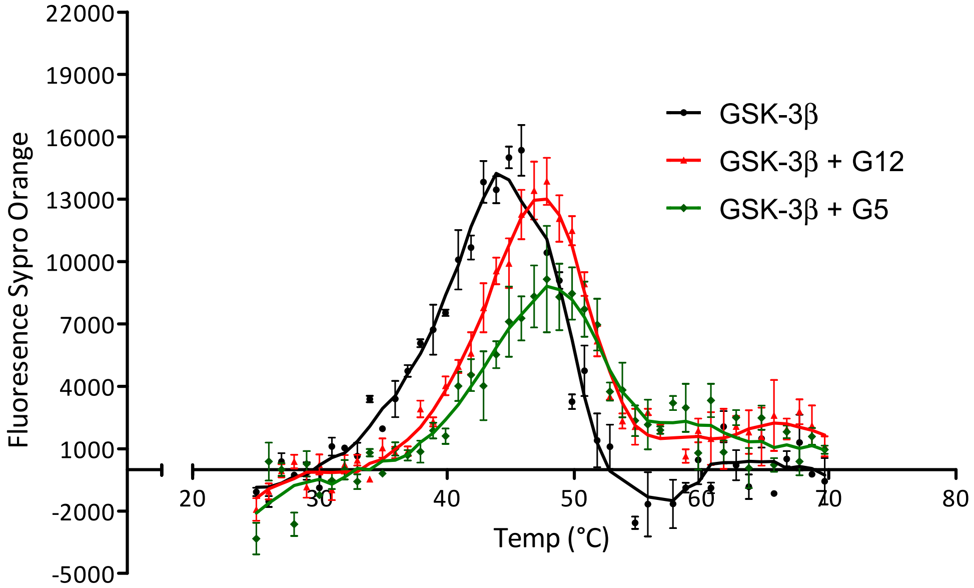

Figure S1. Thermal Shift Assay of GSK-3β alone (black trace), in the presence of G5 (green trace) and G12 (red trace) compounds. G5 and G12 stabilize GSK-3β protein and lead to an increase in melting temperature (T_m) of 3.2 °C and 2.5 °C, respectively. Error bars denote Standard Deviation (SD).

Supplementary Figure S2

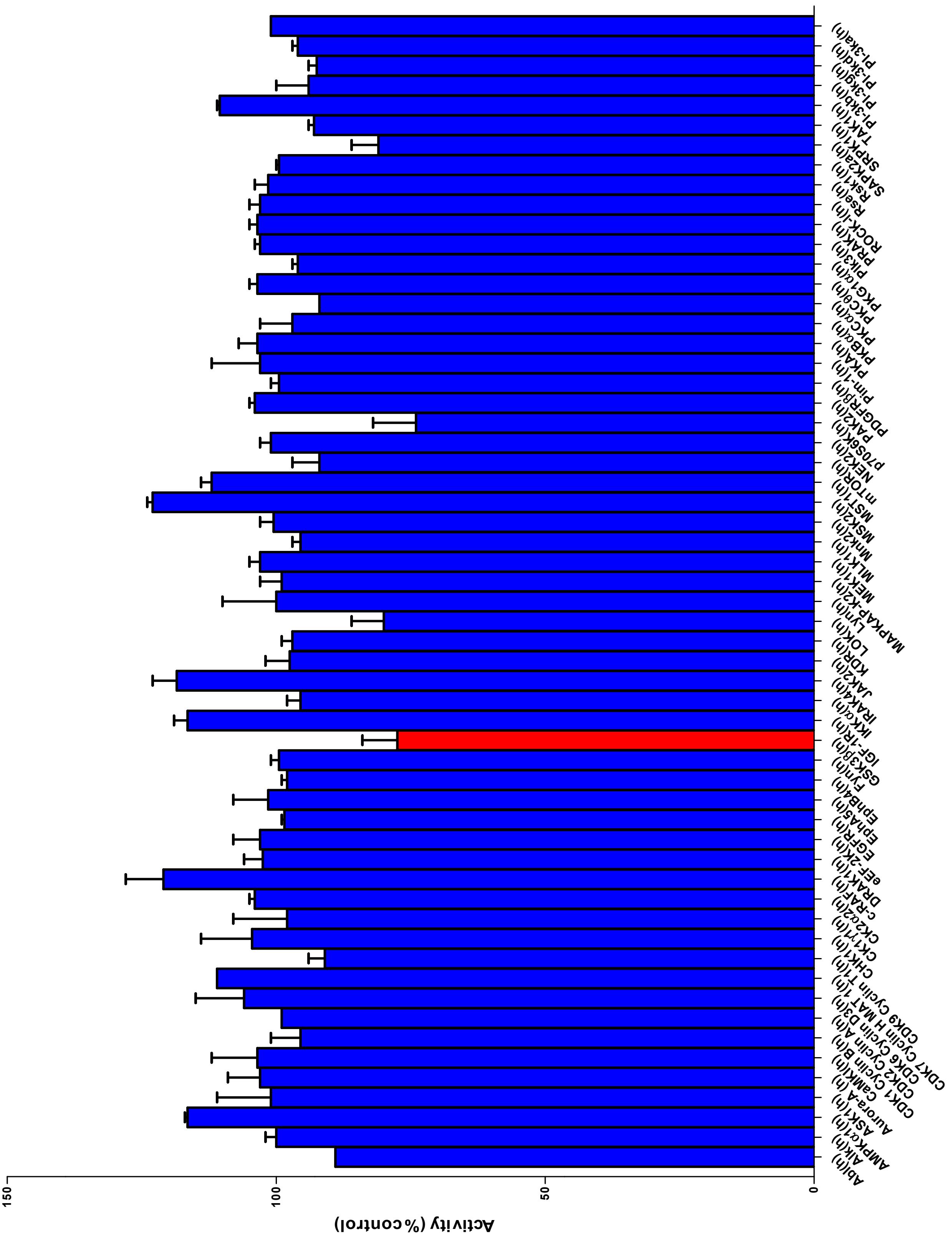

Figure S2. Bar Graphs showing G5 inhibitory effect on different kinases of the commercial panel (Eurofins service); all the experiments were performed using 20 μM of compound and ATP concentrations ± 15 μM of each K_{m}. Error bars denote SD.

Supplementary Figure S3

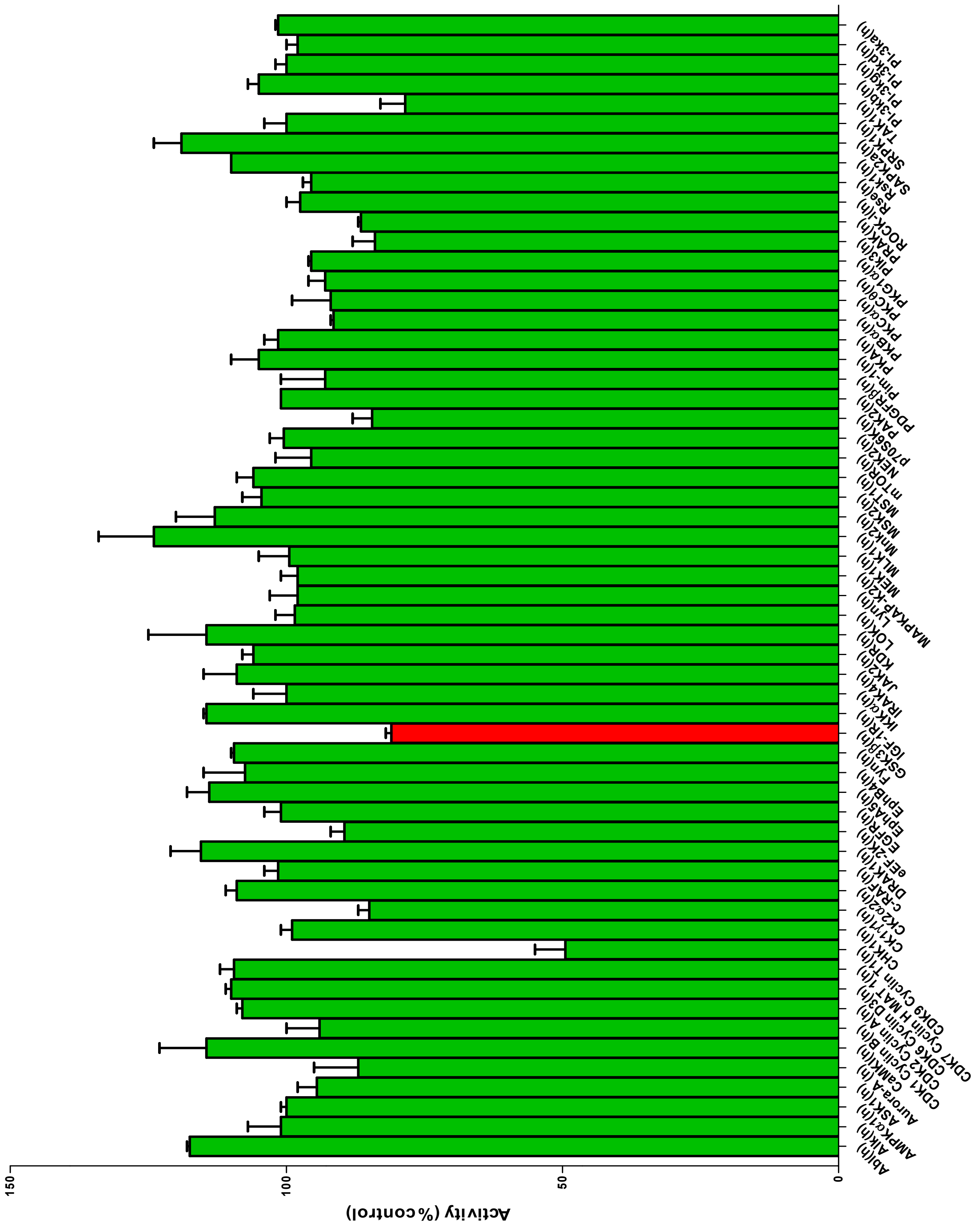

Figure S3. Bar Graphs showing G12 inhibitory effect on different kinases of the commercial panel (Eurofins service); all the experiments were performed using 20 μM of compound and ATP concentrations ± 15 μM of each K_{m}. Error bars denote SD.

Supplementary Table

	**G5**	**G12**
**Data reduction**		
**Resolution (Å)**	2.5-48.8 (2.5-2.59)	2.2-89 (2.2-2.26)
**Rmerge**	0.388 (3.619)	0.319 (3.86)
**Mean(I/sd(I))**	7.9(0.8)	6.9 (0.6)
**Completeness**	100(100)	95.6 (87.8)
**Unique Reflections**	44494 (4582)	61582(4107)
**Multiplicity**	13.4 (12.9)	12.2(11.4)
**$CC_{1/2}$**	0.992 (0.39)	0.993(0.37)
**Refinement**		
**Resolution (Å)**	2.5-48.8	2.2-89
**Rwork/Rfree**	0.225/0.268	0.24/0.27
**RMS bonds (Å)**	0.0086	0.0075
**RMS angles (º)**	1.808	1.573

Table S1. Table reporting refinement statistics and values used for solving structure of GSK-3β in complex with G5 and G12 compounds
